# Pharmacists’ Interventions in Virtual Diabetes Clinics: Cost-Effectiveness Feasibility Study

**DOI:** 10.3390/healthcare13172130

**Published:** 2025-08-27

**Authors:** Sinaa Al-Aqeel, Alaa Mutlaq, Njood Alkhalifa, Deem Alnassar, Rashed Alghanim, Wafa Algarni, Sultanah Alshammari

**Affiliations:** 1 Department of Clinical Pharmacy, College of Pharmacy, King Saud University, Riyadh 11451, Saudi Arabia; 2 General Administration of Pharmaceutical Care, Ministry of Health, Riyadh 11451, Saudi Arabia

**Keywords:** diabetes mellitus, telemedicine, telepharmacy, pharmaceutical services

## Abstract

**Background:** Telepharmacy, the provision of patient care services by pharmacists through the use of telecommunications technology, is associated with improved diabetes-related outcomes and access to healthcare. The primary aim of this study was to characterize pharmacists’ interventions at a virtual pharmacist-led diabetes clinic (PLDC). The secondary aim was to assess the feasibility of conducting a future cost-effectiveness study of the PLDCs. **Methods:** This prospective observational feasibility study was conducted within a pharmacist-led clinic at Seha Virtual Hospital, Riyadh, Saudi Arabia. Two intern pharmacists collected data between 31 July 2024 and 31 January 2025. **Results:** Seventy-five patients (mean [SD] age 50.47 years [14.95]) attended the clinic. The majority were female (58.7%), had type 2 diabetes (86.6%), and were from outside Riyadh (97.3%). The communication with patients was carried out mainly via telephone (73, 97.3%). The mean consultation duration was 7.64 min (SD = 5.68). A total of 179 interventions were conducted, with a mean number of interventions per patient of 2.5 (median 3, min 0, max 5). The most common intervention was patient education and counseling about their disease and medications. While it was feasible to capture the details of pharmacist interventions and resource use data, incomplete data on patient outcomes presented a challenge. **Conclusions:** Our detailed documentation of pharmacist–patient encounters revealed the ability of pharmacists to identify and manage the problems of diabetes patients at virtual PLDCs. Our feasibility study identified a few challenges that need to be addressed when designing future cost-effectiveness studies.

## 1. Introduction

In 2021, approximately 529 million people were living with diabetes worldwide, and more than 1.31 billion people are projected to have diabetes by 2050 [1]. An estimated 37.8 million total diabetes-related years of life were lost and 41.4 million years lived with disability in 2021 [1]. The total cost of diagnosed diabetes in the U.S. was estimated to be nearly USD 412 billion in 2022 [2].

Pharmacists play an important role in diabetes care management and positively impact diabetes-related morbidity and mortality [3,4,5,6,7,8,9,10]. Telepharmacy is the provision of patient care services by pharmacists through the use of telecommunications technology [11]. Cao et al. [12] performed a systematic review on the effectiveness of telepharmacy for patients with diabetes and determined that telepharmacy was associated with a reduction in glycated hemoglobin (HbA1c) and the risk of hypoglycemia. Another systematic review [13] revealed that, compared with usual care, telepharmacy results in similar or better outcomes in patients with diabetes.

Although research on the effectiveness of telepharmacy has reported promising findings, its cost-effectiveness remains uncertain [14,15]. Given this evidence gap, policy-makers lack the information required to make decisions on the funding and reimbursement of telepharmacy services. Cost-effectiveness, sustainability, and reimbursement agreement are significant challenges that e-health and telemedicine implementation face [16,17].

A key strategic objective of the Saudi Health Sector Transformation Program is to increase access to healthcare services. As evidence for telehealth effectiveness has accumulated [18,19], the Saudi Ministry of Health (MoH) has supported the use of innovations, e-health services, virtual care, and digital solutions to improve healthcare accessibility. The Seha Virtual Hospital (SVH), launched in February 2022, is a MoH hospital that facilitates access to specialized virtual healthcare for all regions of Saudi Arabia. It supports 170 hospitals around the Kingdom and offers more than 30 specialized clinics with a capacity of more than 400,000 patients annually [20]. One of these clinics is the pharmacist-led diabetes and internal medicine clinic, which started in November 2023.

Few Saudi studies have examined the effectiveness of face-to-face pharmacist-led diabetes clinic (PLDC) using a nonrandomized study design [21,22,23]. One study evaluated the cost-effectiveness of virtual integrated care clinics for patients with diabetes during the COVID-19 pandemic, with pharmacists being one member of the team, compared with a traditional care model before the pandemic [24]. Thus, local evidence on the effectiveness and cost-effectiveness of telepharmacy services for diabetes patients is lacking.

The primary aim of this study was to characterize pharmacists’ interventions in a virtual PLDC. The secondary aim was to assess the feasibility of conducting a future cost-effectiveness study of the PLDCs through four objectives: estimating eligibility, recruitment, and follow-up rates; assessing the feasibility of collecting outcome and cost data; identifying barriers and facilitators for the service provision; and evaluation and refinement of the data collection form. The design of the feasibility study was in accordance with relevant guidelines [25,26].

## 2. Materials and Methods

### 2.1. Study Design

This was a prospective, observational, feasibility study. The observational nature of the study enabled us to characterize interventions using real-world processes and data without any changes to the pharmacist’s routine patient management.

### 2.2. Setting and Participants

This study was conducted within a pharmacist-led diabetes and internal medicine clinic at SVH. Virtual care at the clinic was offered to patients one day a week, for four hours. Two senior clinical pharmacists alternated running the clinic each week. Clinical pharmacists in Saudi Arabia have postgraduate year 1 (PGY1) with 2 years of experience, postgraduate year 2 (PGY2), or a master’s degree in Clinical Pharmacy with 2 years of experience. All diabetic patients referred to the clinic were eligible.

### 2.3. Data Collection

A Google Form-based data collection form was developed based on previous tools [27,28,29]. The form collected data on patient characteristics and problems as well as pharmacist interventions to address the identified problems. Our data collection form was developed using several previously published, comprehensive, and validated tools. It integrates principles from a tool designed to improve the characterization and consistent reporting of clinical pharmacy services [27], an internationally recognized system for classifying drug-related problems [30], and a comprehensive tool for assessing the clinical and economic impact of pharmacists’ interventions [28]. Two intern pharmacists collected data between 31 July 2024 and 31 January 2025.

### 2.4. Outcome Measures

The outcomes of our study’s primary aim were characteristics of patients referred to the clinic, types of drug-related problems, and the components of pharmacist interventions.

The outcome of our study’s secondary aim, the feasibility study, was eligibility, recruitment, and attendance rates and the feasibility of collecting outcome and cost data. The eligibility rate was calculated as the number of eligible patients relative to those screened. The recruitment rate was estimated as the number of patients recruited per month. The attendance rate was calculated as the proportion of virtual consultations completed as scheduled. For the outcome measure, we assessed the feasibility of collecting HbA1c values before and after the intervention during the study period. For cost data, we assessed the feasibility of identifying resource items used during consultation and measuring the quantity of resources used (volumes).

The cost-effectiveness analysis adopted the perspective of the MoH SVH. The comparator to PLDC is usual care. We assumed that usual care involved no pharmacist intervention; hence, no additional costs for pharmacist time or patients’ transportation were estimated. For the analysis, we planned to estimate the incremental cost-effectiveness ratio (ICER) by dividing the difference in costs by the difference in in HbA1c reduction observed.

### 2.5. Data Analysis

Variables are described as numbers with percentages (n, %) or means with standard deviations (SDs). As a feasibility study that did not aim to assess intervention effectiveness against statistical criteria, a power calculation was not conducted, but we aimed to recruit 70 participants in line with previous recommendations [26].

## 3. Results

### 3.1. Patient Characteristics

During the study period, 75 patients had virtual consultations. The mean patient age was 50.47 years (SD = 14.95). The majority of patients had type 2 diabetes (86.6%), were admitted to the clinic for the first time (65.3%), and were from outside Riyadh (97.3%) (Table 1).

### 3.2. Characterization of Pharmacists’ Activities and Interventions

**Figure 1:**
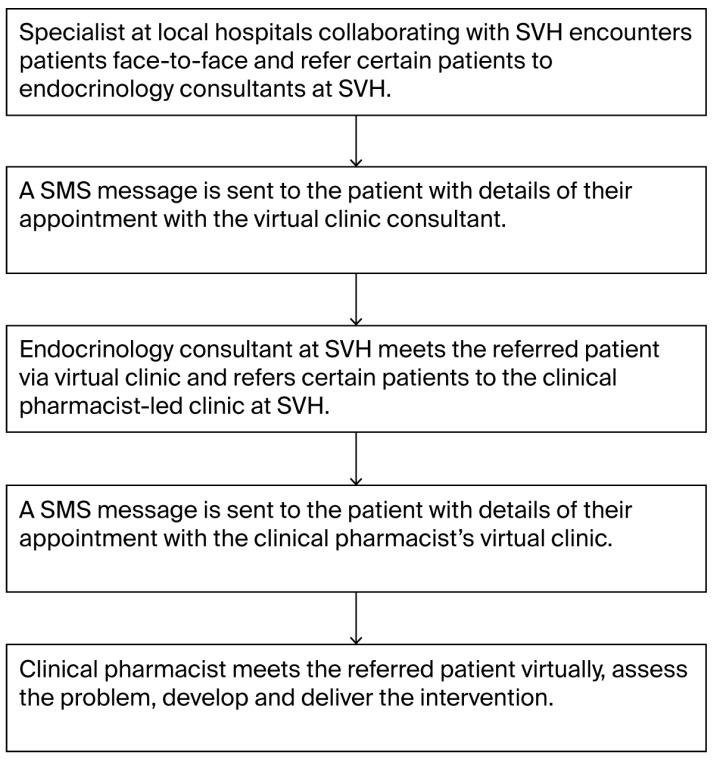
PLDC workflow.

We described the components of pharmacist interventions using the Descriptive Elements of Pharmacist Intervention Characterization Tool [27]. Contact between the pharmacist and patient occurred in a private and individual manner. The recipient of the intervention was the patient or a caregiver (n = 6). The workflow of the clinic is depicted in Figure 1. Patients were referred to the PLDC because of uncontrolled HbA1c, polypharmacy, the addition of new medicine, suspected nonadherence, and the ability to update the patient’s medication list. These patients were provided with the service through a scheduled appointment via telephone (73, 97.3%) or video conference (2, 2.6%) based on the patient preference. For two patients, the consultation started as a video conference, but for technical reasons, it was changed to telephone. The pharmacist obtained information for patient assessment from a digital health records platform and from patients. To construct appropriate interventions, pharmacists started by updating the patients’ medication list to assess the appropriateness of drug indications, contraindications, duplicated drug therapy, and medication interactions. Then, the pharmacists evaluated medication effectiveness (69, 92.0%) and safety (53, 70.6%) by evaluating patients’ symptoms and fasting glucose and HbA1c levels. In 89% of the consultations (n = 67), the pharmacists assessed adherence by asking the patients if they took their medications, at what time, and if they adjusted doses according to blood glucose readings. On a few occasions (n = 13), the pharmacists asked the patients to take photographs of their medications or home blood glucose test readings and send them via WhatsApp.

According to a published tool [30] to classify drug-related problems and identify the causes of these problems, all patients lacked optimal treatment effects, as demonstrated by their uncontrolled HbA1c levels. Based on pharmacists’ discussions with the patients, the cause of this problem was related to the patient’s behavior, specifically inappropriate antidiabetic medication doses (too low, too high); not taking the medications at all; inappropriate timing or dosing intervals; and unhealthy lifestyles. Few patients had problems related to treatment safety, as demonstrated by duplicate therapy (n = 2) and medication side effects (n = 4).

In total, 179 interventions were conducted. The mean number of interventions per patient was 2.5 (median 3, min 0, max 5). The most common intervention was patient education and counseling about their disease and medications (Table 2).

The impact of interventions was evaluated by two raters using the Clinical, Economic and Organization (CLEO) tool [28]. The raters had a high agreement rate (98%). Discussion was required to reach a consensus for 4 of the 179 interventions. The impact was evaluated as a potential minor clinical impact defined as the fact that the intervention can improve knowledge, satisfaction, and medication adherence or prevent harm. The majority of interventions did not change the cost of healthcare, but 49 interventions (27.5%) potentially increased costs due to the ordering of tests, referrals, or recommending a medication.

The mean consultation duration was 7.64 min (SD = 5.68, min = 1, max = 30). After each visit, the pharmacists entered free-text into the database containing a short summary about the patient’s problem and any interventions or recommendations made to be shared with the physicians and other healthcare team members (five minutes per patient). This totaled 15.8 h (9.55 consultations and 6.25 documentations) across 23 clinics.

### 3.3. Feasibility of Cost-Effectiveness Evaluation

The rate of eligibility was 83.3%, as 16.6% of the 90 patients who attended the clinic were not diabetes patients and were consequently excluded. The average rate of recruitment was 21 patients per month (Table 3). Forty-one patients did not answer the phone call (missed appointment).

The collection of outcome measures was challenging. For many patients, no laboratory results for HbA1c were available through the telehealth platform. Among the follow-up patients (n = 26) who attended the first appointment before the data collection period, only two patients had HbA1c values before and after the intervention. Pharmacists asked patients about their HbA1c levels to assess their understanding of the test’s meaning and their target goal. Forty-one patients (55%) reported knowing their last HbA1c reading. The mean self-reported HbA1c value was 9.91 (SD = 1.69).

The collection of resources used and volume was feasible. The main resource item was the pharmacists’ time, which included the time required to read the referral, the virtual consultation time, and the time needed to document the clinic recommendations. The pharmacist’s recommendations, such as ordering a lab test, recommending the addition of a medication, and referring patients to other clinics or healthcare practitioners, are another type of cost. To attach value to the resources used, we multiplied the hourly rate of employing a pharmacist by the visit time. The hourly rate of employing a pharmacist at the midpoint of the salary scale was calculated based on the monthly salary of SAR 20,500 (128 per hour) from published data. The cost of pharmacist consultation per patient was SAR 269 (SD = 121, median 234, min 127, max 745). The total cost per patient, including recommendations such as ordering a test or referral, was SAR 370 (SD 177, median 333, min 127, max 865).

The clinic utilizes a digital platform for health practitioners to schedule patient appointments and provide counseling and a cloud-based digital health record for documenting patient progress notes. The costs of these telehealth platforms include setup, maintenance, software licensing, and internet connection. The clinic is equipped with a computer, headphones, microphones, and telephone. The clinic, including equipment, is not exclusive to the PLDC. These shared costs and fixed costs such as electricity are relevant from the perspective of the MoH; however, the costs attributed to PLDC patients are very small. Furthermore, these costs will be maintained even if PLDCs are terminated. Therefore, these costs were excluded from this study.

We originally planned to calculate ICER using the collected data. However, the incomplete data on pre- and post-HbA1c made this calculation unfeasible. As a result, we could not perform this analysis.

## 4. Discussion

In this descriptive feasibility study, we collected data prospectively from 75 patients who received pharmacist counseling virtually. Pharmacists were able to identify problems and make 179 interventions. The most common intervention was patient education and counseling about their disease and medications. Our secondary aim was to assess the feasibility of conducting a future cost-effectiveness study of PLDCs. It proved feasible to capture the details of pharmacist interventions using an observational study design. The data collection form was comprehensive and easy to fill out. Overall, while collection of relevant costs proved to be feasible, outcome measurement was not feasible and remained a challenge to be addressed.

Our findings concerning the reasons for referral and the content of patient education are consistent with those of Salhia et al. [31] who investigated the reasons for the referral of patients to Saudi MOH medication counseling clinics. During their study period, from May 2020 to December 2021, 28,998 patients were referred to pharmacists counseling either face-to-face (64.55%) or virtually (35.45%), with common reasons for referrals being patients with chronic diseases (50.84%), new medication addition (33.69%), or polypharmacy (22.71%). During the counseling session, the subjects discussed medication name, indication, dose and administration (85.62%), duration of therapy (68.42%), what to do if a dose was missed (44.51%), storage (39.69%), and lifestyle modifications (32.90%).

The types of interventions documented in our study are in concert with those of a previous systematic review [7,10] that reported that the most common pharmacist interventions for patients with diabetes were counseling and education on diabetes, medication, lifestyle modification, and self-monitoring; reinforcement of medication adherence or complication screening; adjustment of pharmacotherapy; and referrals to other healthcare professionals.

Forty-one patients (31%) did not answer the phone and consequently missed appointments. Missed appointments result in unused pharmacist time and decreased efficiency. Our rate of missed appointments was higher than that reported in the literature [32,33]. A UK study of outpatient services revealed that the rate of missed first appointments for remote consultations was higher than that of in-person appointments (12.5% vs. 9.2%), but follow-up appointments had similar rates [32]. The cited reasons for missed in-person appointments include competing work or family/childcare commitments and patients forgetting the appointment [34], and these reasons could be the same for virtual consultations. Future studies could identify individuals at risk of missing virtual PLDC appointments to help the development of interventions to mitigate this risk to help improve efficiency and avoid health inequalities.

Owing to incomplete data on pre- and post-HbA1c, it was not feasible to calculate the extra cost per extra 1% HbA1c reduction in order to decide whether investing in the virtual clinic was an efficient use of resources. A metanalysis [12] reported that telepharmacy is associated with a mean HbA1c reduction from baseline in observational studies (−1.55%; 95% CI, −2.07% to −1.02%) and in randomized controlled trials (−0.86%; 95% CI, −1.25% to −0.47%). However, the heterogeneity between included studies in the design of telepharmacy services make it inappropriate to extrapolate the findings to the SVH clinic.

Lack of access to patients’ medical records and laboratory results was the main barrier to the provision of pharmaceutical services at the virtual PLDCs. Another challenge was the good proportion of patients who did not answer the scheduled phone call. These missed appointments lead to wasted time for pharmacists, the underutilization of resources, and potential delays in care for other patients.

### 4.1. Practice Implications

Our observations indicated that the current pharmacists’ free-text note documentation missed many interventions identified by our data collection form. A study reported that electronic health record documentation of intensive care pharmacists’ interventions increased the capture of interventions by 56% compared with progress notes entering [35]. We recommend replacing free-text documentation with a checklist that captures details of pharmacists’ interventions to capture data fully and reflect the added value of pharmacists’ interventions accurately.

The pharmacist at SVH does not have access to the patients’ medical records at their local hospital. This lack of access compromises the ability of pharmacists to identify drug-related problems, make appropriate recommendations, and optimize medication therapy management [36]. The recent introduction of a shared clinic, where pharmacists and physicians from the local hospital attend the virtual clinic, could be part of the solution to this problem. The shared clinic offers the advantage of allowing pharmacists at SVH to obtain relevant clinical data and enables physicians to approve or disapprove their recommendations instantly. Access to patients’ medical records, or integrating laboratory results and medication lists into the cloud-based digital health record platform, is recommended.

A high missed appointment rate reduces both access to care and provider productivity. The change in clinic time from morning to afternoon and sending a reminder message one day prior to the clinic may decrease the number of missed scheduled appointments. A Saudi study [37] reported that diabetic patients’ attitudes, such as perceived privacy and usefulness of digital health services, significantly impact the perceived intention to use these services. Therefore, policy-makers, physicians, and pharmacists should develop strategies to increase patients’ awareness of the benefits of pharmacists’ consultations and address any concerns about privacy, trust, and ease of use.

The delivery of interventions can be improved if pharmacists engage patients in setting goals and actions for their planned behaviors, such as monitoring glucose blood levels, which have been shown to increase the likelihood of positive outcomes from pharmacist-led interventions [13,38,39]. Pharmacists have identified few cases of nonadherence and addressed the reasons behind it, such as the fear of side effects without the use of a standardized adherence tool. In the future, pharmacists may use tools to measure adherence levels, identify barriers and develop tailored interventions, although selecting a tool for clinical practice is not straightforward [40].

### 4.2. Future Research Recommendations

Future economic evaluations, whether performed alongside clinical trials or through model-based analyses, are warranted. Randomized controlled trials or observational studies conducted in a foreign country may not be able to inform policymaking in the Saudi context due to differences in clinical practices and healthcare financing systems. Therefore, we recommend local trials to assess the impact of virtual PLDCs on outcomes.

When planning a future cost-effectiveness study, access to and the use of patients’ information in local hospitals need to be factored into the study design. For chronic conditions, a long-term time frame is crucial to accurately assess cost-effectiveness by capturing all relevant costs and benefits. For example, in a previous study, one event of non-severe hypoglycemia and one event of severe hypoglycemia were estimated to cost the Saudi MoH SAR 750 and SAR 1712, respectively [41]. Model-based economic evaluations integrate evidence from a variety of sources, including individual trials, meta-analyses, and expert opinion and account for long-term costs and benefits. Our feasibility study focused on assessing HbA1c reduction, as this is associated with lower overall and diabetes-related healthcare costs [42]. Further economic evaluations assessing long-term outcomes, such as mortality, complications, and quality-adjusted life years, are recommended.

Of the 131 patients scheduled for the clinic, 31% did not answer the phone call (missed appointments). Future research should investigate the reasons for missing virtual appointments. One reason could be that elderly patients often provide their caregivers’ contact information, such as a son’s or daughter’s phone number, for scheduling and appointment reminders. As a result, all future communications, including reminders and virtual clinic calls, will be directed to the caregiver. If the caregiver is at work, they may not be available to assist the patient with their virtual appointment. Future work is needed to identify the support required to promote equitable access to care for all patients.

The majority of patients (97%) were from outside the capital city Riyadh, which suggests that the pharmacist was able to provide services to patients who might have otherwise missed them, thereby increasing access to services. However, an exploration of the virtual PLDC’s impact on accessibility was beyond the scope of our investigation. Therefore, a future study specifically focused on accessibility is recommended to determine whether the virtual PLDC facilitates access to specialized pharmaceutical services for all regions of Saudi Arabia.

In our study, four patients were scheduled for the virtual PLDC via video conference, but two were switched to telephone due to technical issues. This suggests the need for further research to explore the impact of technological barriers on service delivery. The impact of technological disparities among different age groups, particularly older adults, on access to the service is a topic for future research.

Future surveys of the opinions and experiences of virtual PLDC users and their willingness to use the service again are needed. To develop a full picture of the pharmacists’ intervention impact, additional studies will be needed that assess physicians’ acceptance of pharmacists’ recommendations.

### 4.3. Limitations

This study had several limitations. Our findings are constrained by data availability and quality. We did not assess the feasibility of assessing long-term outcomes, such as mortality, complications, or quality of life. As our study did not aim to investigate intervention effectiveness, a larger, adequately powered pragmatic study is needed to evaluate effectiveness. While pharmacists can directly make the majority of interventions (79%), actions such as ordering lab tests or starting/stopping medications require physician approval. Because our study did not measure physicians’ acceptance of these interventions, this represents a major limitation.

## 5. Conclusions

Our detailed documentation of pharmacist–patient encounters revealed the ability of pharmacists to identify and manage the problems of diabetes patients at virtual PLDCs. While the collection of relevant costs proved to be feasible, measuring outcomes based on pre- and post-intervention data was not. Our feasibility study identified a few challenges that must be addressed when designing future cost-effectiveness studies.

## Figures and Tables

**Table 1:** Patient characteristics.

	n	%
Age (years)		
<50	39	52.4
≥50	35	46.6
Sex		
Male	31	41.3%
Female	44	58.7%
Appointment nature		
New	49	65.4%
Follow-up	26	34.6%
Region		
Riyadh	2	2.7%
Outside Riyadh	73	97.3%
Type of diabetes		
Type 1	10	13.4%
Type 2	65	86.6%

**Table 2:** Pharmacist interventions.

Type of Intervention	n (%)
Provision of drug information or patient counselling including:	60 (34)
Medication name, doses, and time of administration (with or without food)	46
The importance of lifestyle modification (exercise, weight loss, diet advice)	35
The importance of adherence to prevent complications	24
Self-monitoring blood glucose and target level	23
The importance of screening for diabetic complications (e.g., eye check)	10
Managing acute complications (e.g., hypoglycemia symptoms and management)	7
Medication storage and stability/insulin storage and stability	5
Pregnancy complication	1
Drug monitoring (order laboratory test)	28 (16)
Update of patient’s medication list	26 (15)
Referral to other healthcare professionals or clinics	21 (12)
Dose adjustment	19 (11)
Recommend discontinuation (suspend prescription)	6 (3)
Administration mode optimization	6 (3)
Monitoring results report	7 (4)
Recommend addition (new prescription)	3 (2)
Help with refill procedures	3 (2)

**Table 3:** Rate of eligibility and recruitment.

	Answered the Call (n = 90)		Did Not Answer the Call (n = 41)	Total
Month	Eligible	Excluded		
July	2	0	0	2
August	6	0	1	7
September	11	5	4	20
October	14	3	9	26
November	9	6	9	24
December	14	1	8	23
January	19	0	10	29
Total (%)	75 (57.3)	15 (11.4)	41 (31.3)	131

## Data Availability

The original contributions presented in this study are included in the article. For further inquiries, please contact the corresponding author.

## Abbreviations

The following abbreviations are used in this manuscript:

MoH	Saudi Ministry of Health
PLDC	pharmacist-led diabetes clinic
ICER	cost-effectiveness ratio
